# Book Reviews

**Published:** 1897-12

**Authors:** 


### Book Reviews.

---

Tuberculosis of the Genito-Urinary Organs, Male and Female, By N. Senn, M. D., LL. D., Professor of Practice of Surgery and Clinical Surgery, Rush Medical College; Attending Surgeon to Presbyterian Hospital; Surgeon-in-Chief St. Joseph's Hospital, Chicago, Octavo, pp. vi.–317. Illustrated. Philadelphia: W. B. Saunders, 925 Walnut street. 1897.

It is only in comparative recent years that tuberculosis of the genital organs has attracted the special attention of the profession. In other words, it may be affirmed that this is, comparatively speaking, a new field of surgical pathology. The work before us is the first within our knowledge to deal with the subject in a separate treatise in a comprehensive manner.

It has been known for some time that it is possible to obtain direct tubercular inoculation through coitus, and Senn points out the fact that where the penis has been amputated for supposed carcinoma, in many instances the disease was really tuberculosis. The latter may be strongly suspected when no recurrence follows the operation. It seems to be a fact that no locality in the male genital tract is free from the liability of tubercular invasion. Thus it may appear in the urethra, in the spermatic cord, in the seminal vesicles, in the prostate, in the testicle and in the epididymis, though in not all as a primary lesion. Senn asserts that it is never primary in the spermatic cord, nor in the prostate, and rarely so in the testicle. Parts I. and II., comprising the first eighty pages of the book, are devoted to this branch of the subject.

The author, in Part III., takes up the consideration of tuberculosis of the female organs of generation, in which he discusses most interestingly the manner of infection, the pathology and the treatment of the malady. Tuberculosis of the vulva is briefly handled in Part IV.; tuberculosis of the vagina is more elaborately considered in Part V., and direct infection by coitus affirmed.

Tuberculosis of the uterus is taken up in Part VI., and receives ample treatment at the hand of this author. The pathology, etiology, symptoms, diagnosis, prognosis and treatment are all dealt with in intelligent sequence. In treatment the only reliable method that promises cure is extirpation. Senn prefers vaginal hysterectomy to the abdominal route, because the former, he says, is attended by less risk of peritoneal infection ; but in the next sentence he affirms that in all these cases it should be taken for granted that the peritoneum is already more or less affected, and advises that the parts in the vicinity of the organ removed should be iodoformised and that vaginal drainage with iodoform gauze should be established. We fail to appreciate the author's preference for the vaginal route in view of the reason given and of the facts stated afterward.

The next two, Parts VII. and VIII., are devoted to the consideration respectively of tuberculosis of the Fallopian tubes and tuberculosis of the ovaries. It is probable that tuberculosis of the tubes is much more common than has heretofore been believed. It is very often not suspected before operation and perhaps quite as frequently undetected afterward. Nevertheless, now and then, tubercle bacilli have been found in pus removed from tubal abscesses. Senn gives credit to Edebohls, Williams and Penrose in this direction. The operative treatment in these cases is unquestioned, but the difficulties of diagnosis are admittedly very great. The ovary is seldom the seat of primary tuberculosis, but when it does exist it is, according to Senn, caused by hematogenetic infection in the same manner as tuberculosis of the testicle.

Tuberculosis of the bladder is the subject of Part IX., and tuberculosis of the ureter forms that of Part X. Chronic cystitis has, no doubt, been mistaken for tubercular disease. Mistakes in diagnosis have led to improper treatment. Senn points these facts out with great cogency. The ureter is rarely the seat of tubercular disease, but occasionally is affected by the processes of extension.

The final section of the book, Part XI., comprising ninety pages, is devoted to the consideration of tuberculosis of the kidney. Here Senn is most interesting, displaying his intimate knowledge of the subject and his masterful methods in diagnosis and treatment. Here, too, is his knowledge of pathology well demonstrated. Some of the illustrations in this section are excellent, especially figure twenty-two, opposite page 234, where subcapsular nodules in a case of miliary tuberculosis are beautifully demonstrated.

The book is handsomely printed, well indexed and must take rank as one of the author's best contributions to the literature of American medicine.

The Origin of Disease, Especially of Disease Resulting from Intrinsic as Opposed to Extrinsic Causes. With chapters on diagnosis, prognosis and treatment. By Arthur V. Meigs, M. D., Physician to the Pennsylvania Hospital. Octavo, pp. xiv.—229. With 137 original illustrations. Philadelphia: J. B. Lippincott Co. 1897.

There is no truer statement than the following from the author's preface: "The advantages of specialism are patent; but it is certain that a wide divergence of knowledge also results from it, and that facts which belong together are often observed by persons whose lines of research lead them so often apart that for a long time the conclusion remains undiscovered. There is great need for something to counterbalance this inevitable evil result, and to bring together the facts which, isolated, are useless. The relation of clinical medicine with pathology is obvious, and there can be no doubt that they are separated to the disadvantage of both." Acting upon this affirmation of fact, Meigs has industriously sought to draw nearer and nearer together the widely divergent lines of clinical medicine and pathology. That he has succeeded to a considerable degree will become apparent to anyone who studiously reads the interesting pages of his book.

Beginning with the diseases of age, to which he devotes a chapter of ten pages, we next find the author dealing with the origin of disease in a chapter of about eighteen pages, from which the book takes its title. This is a most interesting chapter, dealing with generalities which the author reduces to particulars. Next follow chapters on the blood-vessels, heart, lungs, liver, spleen, stomach, intestines, kidneys and spinal cord, in which the pathology of these several systems, organs or viscera is carefully and minutely studied. Then follow chapters on diagnosis, prognosis and treatment in chronic disease.

The value of this work consists chiefly in the fact that the observations of the author have been verified by careful post-mortem examinations, aided by a scientific use of the microscope. The illustrations are of a nature to justify an author's pride. They are all made from tissue sections (from the author's preparations, with two exceptions,) etched on steel, and the relations of the parts have been kept true to nature. Such work as Meigs is doing cannot help but advance the science of medicine and the present book is a great stride in that direction. We look to see it adopted in college instruction.

---

THE DISEASES OF WOMEN. A Handbook for Students and Practitioners. By J. BLAND SUTTON, F. R. C. S., Eng., Surgeon to the Chelsea Hospital for Women; Assistant Surgeon, Middlesex Hospital, London, and Arthur E. Giles, M. D., B. Sc., London, F. R. C. S., Edin., Assistant Surgeon, Chelsea Hospital for Women, London. Small 8vo, pp. 436. Philadelphia: W. B. Saunders, 925 Walnut street. 1897.

This manual departs from the usual custom in that it is written, as announced by the author, with the object of usefulness to the student for examination purposes. The senior author is well known to the professional world as an experienced clinician and writer. Therefore the success of this, his most recent literary venture, would appear to be assured. The junior author, though not as well known, has been for some years assistant to Mr. Sutton at the Chelsea Hospital for Women, hence it is to be presumed that much of the work in preparing this book was performed by Dr. Giles.

One of the first things relating to the book to be commended is the clearness with which the authors set forth the physiology of the reproductive organs. This subject is most difficult for students to understand, but here they will find substantial aid both in text and in illustration to the elucidation of this complex process. The chapters on tubal pregnancy are also written with perspicacity and add much to the value of the manual. Sutton's knowledge of diseases of the Fallopian tubes and ovaries has been heretofore demonstrated and he and his associate have profited by further experience, as indicated in their chapters on those subjects.

The points to which we have alluded are those most enshrouded in mystery and are hence most taxing to the comprehension of the student. It is for this reason that we have dwelt upon them. The book abounds in good material that is most admirably presented and in addition to catching the eye of students it will command the respect and attention of the profession.

Transactions of the American Gynecological Society. Volume XXII. For the Year 1897. J. Riddle Goffe, M. D., Secretary, Philadelphia: Wm. J. Dornan, Printer. 1897.

The annual transactions of this society always appear promptly and in good form. This volume is no exception to the rule, though it is slightly smaller than the last one, but the scientific excellence of the papers is maintained at the previous high standard set by the society. The president, Dr. James R. Chadwick, chose for the subject of his presidential address, A historical sketch of abdominal surgery, which contains many interesting facts with dates that will make it a valuable paper for reference. Dr. Thomas Addis Emmet claimed the privilege of adding a few words in confirmation of many of the facts referred to in the address, because they had transpired within his own experience. He then proceeded to give an account of a futile attempt made to open the abdomen of a young woman in 1855 at the Woman's Hospital. Dr. Sims was opposed by some of the members of the board, Dr. Stevens among the number, and desisted. Again, however, about 1860, the subject came up and Dr. Emmet assisted Dr. Sims to remove a parovarian cyst, but they did not dare to have anyone else present at the operation or ask for a consultation !

The society elected Dr. Paul F. Munde, of New York, to be president for the ensuing year—a fitting recognition of the zeal and ability of the real founder of the society. The next meeting will be held on the fourth Tuesday in May, 1898, at Boston.

REPORT of the Commissioner of Education for the Year 1895-96. Volume I. Containing Part I. Washington: Government Printing Office. 1897.

It is interesting to note that these reports are issuing with greater promptitude than was formerly the case. An instructive chapter in this volume considers laws relating to city school boards, and among the number Buffalo is included. Among other things is mentioned the fact that Buffalo has a board of school examiners consisting of five citizens appointed by the mayor, who hold office for five years each. One is appointed each year. The mayor-elect being a member of this board, Dr. Diehl will have two vacancies to fill when he takes office January 1, 1898. The duties of the board of examiners, inter alia, are to visit and inspect all the schools once in each term and to report their condition to the common council, including therein a statement as to the operation of the system of examinations and any suggestions they deem proper for the more efficient accomplishment of the purposes of their appointment. We have called attention to this fact to show the importance of selecting able and efficient men for school examiners.

This report abounds with information useful to educators and their coadjutors in the cause of educational improvement.

---

The Roller Bandage with a Chapter on Surgical Dressing. By William Barton Hopkins, M. D., Surgeon to Pennsylvania Hospital. Illustrated. Philadelphia: J. B. Lippincott Co. 1897.

It is important that a student of surgery should be taught the correct principles of bandaging early in his career. The first and best way to do this is at the bedside or in the clinic. He should be made to apply bandages under the eye of a competent instructor; he then needs a proper manual for study and reference in the intervals of his practical instruction. Hopkins has supplied just such a manual in this book, which is planned to teach by illustration rather than description. The drawings are made from photographs and have been made to portray the originals with faithfulness. This is the fourth edition of the Roller Bandage, which indicates its usefulness and popularity.

---

Transactions of the Medical Society of the State of West Virginia, held at Charleston, May 19, 20 and 21, 1897. Instituted April 10, 1867.

Though West Virginia is, comparatively speaking, a new state, yet its medical society is thirty years—nearly a generation—old. The titles of the papers published herein indicate a high standard of professional and literary qualifications on the part of the members. Dr. W. H. Sharp, of Parkersburg, discusses the etiology and treatment of eclampsia in an interesting manner, in which he gives the several points of view of prominent authorities. We could wish to see this volume bound in more permanent covers.

---

The American Academy of Railway Surgeons,. Report of the third annual meeting, held at Chicago, Ill., September 23, 24 and 25, 1896. Edited by R. Harvey Reed, M. D., Columbus, O. Chicago: American Medical Association Press. 1897.

In this book is well recorded the proceedings of the American Academy of Railway Surgeons at its third annual meeting, including papers read and discussions held. The Academy makes a creditable showing. This volume contains fifty more pages of reading matter than the last and 150 more than the previous one. This fact shows the continuous and healthy growth of the Academy. Among other illustrations the book contains a fine portrait of Dr. E. M. Dooley, of Buffalo, who was acting-secretary during the meeting at which this book was made and is surgeon of the N. Y., L. E. & W. Railway. The editor is to be congratulated upon the form and manner of the publication of this book of transactions.

---

Annual Report of the State Board of Charities for the Year 1896. Transmitted to the legislature February 25, 1897. Albany and New York: Wynkoop Hallenbeck Crawford Co., State Printers. 1897.

This annual report is always full of interest if not instruction. It is a record of the transactions of one of the best organised charity commissions in the country and it has always been administered by able minds. The power which the law confers upon the board is remarkable. In addition to the duties of inspecting the various charities the board also exercises authority over their expenditures. Its functions are usually exercised in the direction of retrenchment and always, we believe, with due regard to economy. It is especially appropriate, therefore, that when the board recommends that the salary of the superintendent of the Soldiers' and Sailors' Home at Bath shall be increased from $2,000 to $3,000 a year, such suggestion should be promptly complied with, which we believe was done.

---

Essentials of Bacteriology: Being a concise and systematic introduction to the study of microörganisms, for the use of students and practitioners. By M. V. Ball, M. D., Bacteriologist to St. Agnes' Hospital, Philadelphia. Duodecimo, pp. xvi.—218. Third edition, revised. Illustrated. Philadelphia: W. B. Saunders, 925 Walnut street.

The modest title page will describe this little volume as a concise introduction to the study of microörganisms. It will be especially valuable to the older practitioner, who wishes to understand somewhat the methods of the bacteriological laboratory, without going into the subject too deeply, and the student will find it helpful in the early part of his course, when the whole field of medicine is an unknown land. The facts are clearly and accurately stated and the illustrations are good.

